# Supplementary material for: Gene and protein expression and metabolic flux analysis reveals metabolic scaling in liver ex vivo and in vivo
Source: eLife. 2023 May 23;12:e78335. doi: 10.7554/eLife.78335 (PMC10205083; doi:10.7554/eLife.78335)
Supplement: Supplementary file 1. [file elife-78335-supp1.docx]

| **Gene** | **Forward primer** | **Reverse primer** |
| --- | --- | --- |
| Mouse *Actb* | GTGACGTTGACATCCGTAAAGA | GCCGGACTCATCGTACTCC |
| Rat *Actb* | TAC AACCTCCTTGCAGCTCC | GGATCTTCATGAGGTAGTCAGTC |
| Mouse *Glul* | GTTCCCACTTGAACAAAGGCA | ACCCAGATATACATGGCTTGGA |
| Rat *Glul* | TCCAGATAGGACCCTGCGAA | CTGAGATGAAGCGGTGGGTT |
| Mouse *Lipe* | GATTTACGCACGATGACACAGT | ACCTGCAAAGACATTAGACAGC |
| Rat *Lipe* | CTCCAGGAGGCTTGGAACAG | TCTCTCTCCCTAGGCCCAAC |
| Mouse *Dlst* | GGAACTGCCCTCTAGGGAGA | GACGCTACCACTGTTAATGACC |
| Rat *Dlst* | CACCCATTATCAACCCGCCT | TACAGCCAGCCATTTAGCGA |
| Mouse *Mmp3* | TGGGCCCTCAGACGTTTTT | GCAGTGCCTGAGATTCACCT |
| Rat *Mmp3* | TTCTGGGCCCCTCAGAAGT | GGCATGACTCTCACAATGCG |
| Mouse *Larp6* | AGGACTGAGTGTCTGTTCCCT | AAACCATCTAAGCCGCCTGT |
| Rat *Larp6* | TCCGGACGAGGAGCTTATCA | CCACAAGGTCCACACCTCAG |
