# Supplementary material for: Gene and protein expression and metabolic flux analysis reveals metabolic scaling in liver ex vivo and in vivo
Source: eLife. 2023 May 23;12:e78335. doi: 10.7554/eLife.78335 (PMC10205083; doi:10.7554/eLife.78335)
Supplement: Supplementary file 2. — APE indicates the atom percent enrichment (in animals infused with 13C tracer), TCA denotes the tricarboxylic acid cycle, and GNG denotes gluconeogenesis. By convention, Va represents the flux through pathway a. [file elife-78335-supp2.docx]

| **Equation** | **Interpretation** |
| --- | --- |
| 1. ${}^{13}C4 malate=Total {}^{13}{C malate-}{}^{13}{C1C2C3 malate}$ | [4-^13^C] malate, equivalent to [1-^13^C] malate |
| 1. ${}^{13}C2C3 malate=Total {}^{13}{C malate- 2*(}{}^{13}{C4 malate)}$ | Enrichment in carbons 2 and 3 of malate |
| 1. $Turnover=\left( \frac{Tracer APE}{Plasma APE}-1 \right)*Infusion rate$ | Whole-body endogenous glucose or palmitate production |
| 1. $GNG= Glucose turnover$ | Whole-body gluconeogenesis |
| 1. $\frac{V_{PEPCK}}{V_{GNG}}\sim\frac{V_{PC}}{V_{GNG}}=\frac{[{}^{13}{C_{2}]}glucose}{{XFE}^{2}}$ | Fraction of gluconeogenesis derived from pyruvate |
| 1. $XFE=\frac{1}{1+\frac{[{}^{13}{C_{1}]}glucose}{2*[{}^{13}{C_{2}]}glucose}}$ | Fractional triose enrichment |
| 1. $Corrected \left[ {}^{13}{C_{2}} \right]glucose=Measured [{}^{13}{C_{2}]}glucose-2*[C4C5C6-{}^{13}{C_{2}}]glucose$ | Doubly-labeled glucose arising from the condensation of two singly labeled trioses, correcting for doubly labeled glucose arising from one doubly labeled triose condensing with an unlabeled triose |
| 1. $\frac{V_{PC}}{V_{CS}}=\frac{\left[ 5-{}^{13}C \right]glucose}{2*\left[ 4-{}^{13}C \right]glucose}$ | Rate of pyruvate carboxylase anaplerosis relative to TCA cycle flux |
| 1. $\frac{V_{PDH}}{V_{CS}}=\frac{\left[ 4-{}^{13}C \right]glutamate}{\left[ {}^{13}C \right]alanine}$ | Fractional contribution of glucose to the TCA cycle |
| 1. $V_{PC}=\frac{V_{PC}}{V_{GNG}}*V_{GNG}$ | Absolute rate of gluconeogenesis from pyruvate |
| 1. $V_{CS}=\left( \frac{V_{PC}}{V_{CS}} \right)^{-1}*V_{PC}$ | Absolute TCA cycle flux |
| 1. $V_{PDH}=\frac{V_{PDH}}{V_{CS}}*V_{CS}$ | Absolute rate of glycolytic carbon entry into the TCA cycle |
| 1. $V_{FAO}=V_{CS}-V_{PDH}$ | Absolute rate of entry of carbons from oxidized fatty acids into the TCA cycle |
